# A Model and Cheap Ice House

**Published:** 1854-12

**Authors:** 


### A MODEL AND CHEAP ICE HOUSE.

A correspondent of the New England Farmer gives a description of a very cheap mode of erecting an ice house, which he has found to keep ice the year round without any difficulty, and the cost of which does not exceed twenty-five dollars. The following is the description of his mode of building:

In the first place, dig out the dirt where you wish your house to stand, to the depth of two feet, or more, if exposed to frost greatly, and fill up with stone, then put your sills on level with the ground, put in strong sleepers, and cover this over with three-inch plank. Commence upon these plank with scantling, sawed one and a half by four inches for your wall, laying one upon the other and nailing them one to the other; inside of this lay up another tier, leaving a space of about four inches to be filled up with sawdust, or tan-bark; so continue till you have it as high as you wish for ice; then take plank and cover over, having them to the outer edge of your inside wall; continue on as before with your walls, until as high as you like, and cover all over with plank two inches double; if under, no roof will be necessary more than this. Have two doors, one where the ice is, and one above where you can put in butter, milk, or anything you like to have kept cool. Bore these upper plank full of two-inch holes, the ones above the ice; and the ones at the bottom of the house bore half-inch holes through them once in about a foot all over the bottom, and spread two inches or more of sawdust or tan-bark over the bottom before putting your ice in. Pack the ice as closely as possible, not having it come quite up to the top and edge of your house, and when full throw sawdust, or whatever you may use, over the top, and also fill up between the ice and sides of the house.

